# Epidemiology of the lymphatic-dwelling filarioid nematode *Rumenfilaria andersoni* in free-ranging moose (*Alces alces*) and other cervids of North America

**DOI:** 10.1186/s13071-016-1740-x

**Published:** 2016-08-12

**Authors:** Caroline M. Grunenwald, Michelle Carstensen, Erik Hildebrand, Jacob Elam, Sauli Laaksonen, Antti Oksanen, Richard W. Gerhold

**Affiliations:** 1 Department of Microbiology, University of Tennessee, M409 Walters Life Sciences, Knoxville, TN 37996-0845 USA; 2 Minnesota Department of Natural Resources, 500 Lafayette Road, St. Paul, MN 55155-4040 USA; 3 Department of Veterinary Biosciences, University of Helsinki, Fabianinkatu 33, FI-00014 Helsinki, Finland; 4 Finnish Food Safety Authority Evira, Production Animal and Wildlife Health Research Unit (FINPAR), Elektroniikkatie 3, FI-90590 Oulu, Finland; 5 Department of Biomedical and Diagnostic Sciences, College of Veterinary Medicine, University of Tennessee, 2407 River Drive, Knoxville, TN 37996 USA

**Keywords:** *Rumenfilaria andersoni*, Cervids, Lymphatic filariasis, Bioinvasion, Parasite translocation, Moose (*Alces alces*), White-tailed deer (*Odocoileus virginianus*)

## Abstract

**Background:**

Moose (*Alces alces*) are a culturally and economically valued species in Minnesota, where the northeast population has decreased by 60 % since 2006. The cause of the decline is currently unclear; however, parasites, predation, and climate change have all been implicated. Nematode parasites are important pathogens in North American moose, potentially causing severe disease and mortality. Recent spread of *Rumenfilaria andersoni*, a filarioid nematode of moose, has been documented in Finnish cervids; however, little is known about the epidemiology of this parasite in North America.

**Methods:**

To investigate the prevalence and distribution of *R. andersoni*, 584 blood samples were collected from live-captured and dead animals and screened microscopically for the presence of microfilariae using a modified Knott’s test. Microfilariae were identified based on morphological characteristics. A subset of Knott’s-positive animals was subjected to polymerase chain reaction (PCR) with filarioid-specific primers targeting the first internal transcribed spacer region (ITS-1) of the rRNA gene cluster.

**Results:**

*Rumenfilaria* microfilariae were present in 20.5 % of Minnesota moose (*n* = 352), with slight fluctuations observed over four years. Minnesota white-tailed deer *(Odocoileus virginianus)* (*n* = 2) and moose (*n* = 44) from Alaska, Montana, Washington, Maine, and New Hampshire also harbored *R. andersoni*, suggesting this parasite occurs widely throughout North American moose herds, and white-tailed deer can serve as a patent host. Sequence analysis of cervid blood (moose, *n* = 15*;* white-tailed deer, *n =* 1) confirmed the identity of *R. andersoni* and revealed the existence of two distinct clades. Genetic comparisons of *R. andersoni* isolates from North America and semi-domesticated Finnish reindeer found the two groups to be closely related, supporting previous hypotheses that *R. andersoni* was recently introduced into Finland by the importation of deer from the United States.

**Conclusions:**

To the best of our knowledge these observations represent the first report of *R. andersoni* within the contiguous United States and reveal this nematode as a common parasite of North American moose and white-tailed deer. Although the implications of *R. andersoni* infection on moose health is unclear, increased awareness of this parasite will help prevent unintentional introduction of *R. andersoni* into naïve populations via the translocation of wild and captive cervids.

## Background

Moose (*Alces alces*) have long been a culturally and economically valued species in North America; however, some North American moose populations have exhibited a serious decline in population [1–3]. Nowhere has this decline been more dramatic than in Minnesota, where the estimated number of free-ranging moose in the northeastern region has decreased by 60 %, from 8840 animals in 2006 to 3450 in 2015 [4]. Disease, parasites, predation, and climate change have all been implicated as factors related to the decline [5–9]. To prevent further loss of this species and natural resource, a better understanding of the Minnesota moose herd’s overall health, as well as an understanding of the potential drivers of mortality, is urgently needed.

Nematode parasites, particularly species of lungworms and filarioids, are important pathogens in moose and are known to cause morbidity and mortality in free-ranging populations [6, 10–12]. *Rumenfilaria andersoni* is a lymphatic-dwelling filarioid nematode associated with moose and caribou (*Rangifer tarandus*). The species belongs to the family Onchocercidae, which is a group of filarioid nematodes transmitted by hematophagous arthropods. Although the exact details of the *R. andersoni* life-cycle have yet to be elucidated, all adult filarioids of the family Onchocercidae produce larval stages called microfilariae that reside in the circulatory system of the definitive cervid host. When an arthropod intermediate host (vector) obtains a blood meal and ingests the microfilariae, the microfilariae unsheathe, penetrate the vector’s gut wall, and develop to an infectious larval stage within the vector’s hemocoel. Extrinsic development is completed when infectious larvae are inoculated into a definitive host by the vector during a subsequent blood meal.

Originally described in moose from Ontario [13] and more recently in Alaska [14] and Finland [15, 16], the exact geographical distribution and vector identity for *R. andersoni* are unknown. Although the pathological impact of this filarioid nematode on cervid health remains unclear, recent studies documenting the rapid expansion of *R. andersoni* in Finnish semi-domesticated reindeer (*R. tarandus tarandus*) describe inflammation of ruminal lymphatic vessels and high microfilariae load within the bloodstream [15, 16], both of which are predicted to have a negative impact on overall cervid health [17–19]. These results suggest *R. andersoni* infections may have important health implications for cervids, including moose.

The main objective of this study was to investigate the eco-epidemiology of *R. andersoni* in Minnesota moose compared to other North American herds. To accomplish this, we explored the basic epidemiology of this parasite, including the identification of other patent host species, geographical distribution, prevalence of infection, and host demographics by collecting parasitological samples across spatial and temporal scales. We also performed the first genetic characterization and comparison of *R. andersoni* isolates to provide a basis for future population genetics studies. These data will contribute to a greater understanding of *R. andersoni* biology and provide baseline epidemiological data for future reference and research.

## Methods

### Sampling of hosts and detection of microfilariae

**Fig. 1:**
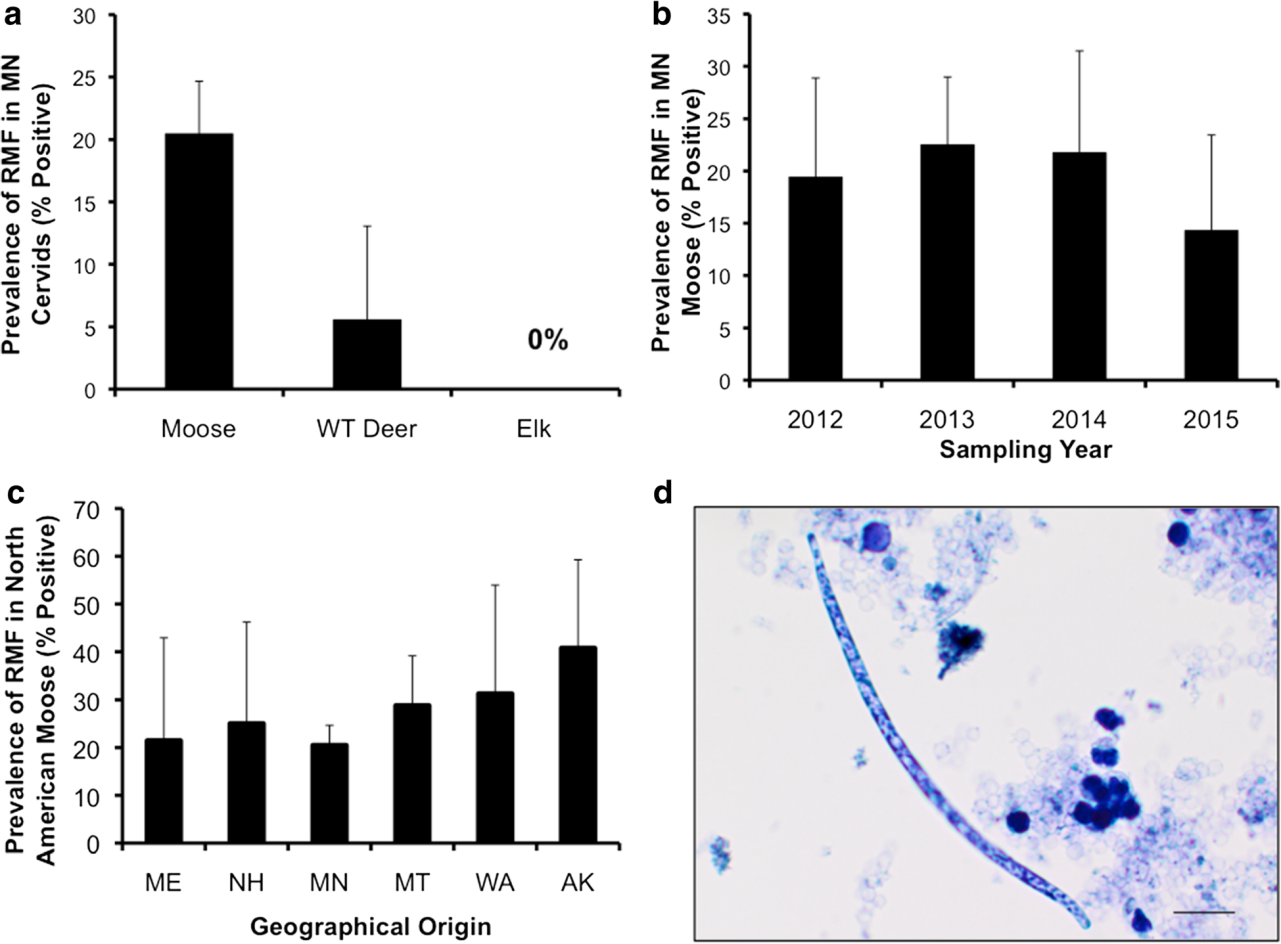
Prevalence of *R. andersoni* microfilariae (RMF) in blood samples drawn from free-ranging cervids. Prevalence is defined as the percentage of samples that tested RMF-positive using a modified Knott’s test. Error bars represent 95 % confidence intervals. **a** Prevalence of RMF in three species of Minnesota cervids (moose, *n* = 352; elk, *n* = 14; white-tailed (WT) deer, *n* = 36). Blood samples were collected over a four-year period for moose and a two-year period for elk and deer. Fisher’s exact test; *P* = 0.013. **b** Comparison of RMF prevalence in Minnesota moose over time (2012, *n* = 67; 2013, *n* = 160; 2014, *n* = 69; 2015, *n* = 56). Fisher’s exact test; *P* = 0.607. **c** Comparison of RMF prevalence in moose from several U.S. states (MN, *n* = 352; NH, *n* = 16; ME, *n* = 14; MT, *n* = 73; WA, *n* = 16; AK, *n* = 27). Fisher’s exact test; *P* = 0.013. **d** Image of RMF from Minnesota moose blood. Sample was stained with methylene blue and viewed with a bright light microscope at 200× magnification. *Scale-bar*: 20 μm

To estimate the prevalence of *R. andersoni* within the Minnesota moose population, 352 blood samples were collected between 2012 and 2015. Blood was collected from either hunter-killed animals, opportunistic mortalities, or live-captured animals and placed into 5-ml EDTA blood tubes. A limited number of blood samples were also obtained from hunter-harvested wild elk *(Cervus canadensis)* (*n* = 14) and live-captured and hunter-harvested free-ranging white-tailed deer *(Odocoileus virginianus)* (*n* = 36) in Minnesota. Additional blood samples were donated by various state wildlife agencies from live-captured and hunter-harvested animals, including 12 caribou and 27 moose from Alaska; 14, 73, and 16 moose from Maine, Montana, and New Hampshire; and 16 moose, 1 white-tailed deer, and 3 mule deer *(Odocoileus hemionus)* from Washington. Whenever possible, the age, sex, and geographical location of the animal was noted. Animals were classified as calves (< 1 year of age), yearlings (1 to < 2 years of age), or adults (≥ 2 years old). Blood samples (1 ml/animal) were refrigerated and shipped to the Molecular Parasitology Laboratory at the University of Tennessee College of Veterinary Medicine to identify the presence of *R. andersoni* microfilariae (RMF) using a modified Knott’s test and bright-light microscopy [20]. Microfilariae were identified based on comparative morphological features [15, 20, 21] (Fig. 1d).

### Prevalence estimates and statistical analyses

Prevalence of *R. andersoni* within cervid populations was estimated for each cervid species based on the modified Knott’s test results. Blood samples were categorized as RMF positive based on the presence of microfilariae with morphological features consistent with *R. andersoni* identified via the modified Knott’s test described above. Animals with no microfilariae present or microfilariae morphologically distinct from *R. andersoni* were categorized as RMF negative. To determine if *R. andersoni* prevalence differed between geographical locations, a Pearson’s *χ*^2^ test or Fisher’s exact test was used with Bonferroni correction (*P* ≤ 0.012). The Minnesota moose population was further analyzed by comparing prevalence between age class, sex, and sample year using the Pearson’s *χ*^2^ test or Fisher’s exact test with Bonferroni correction (*P* ≤ 0.012). Statistical analyses were performed with SPSS software version 23.0 (IBM Corporation, Armonk, NY, USA).

### Molecular confirmation of microfilariae as *R. andersoni*

To confirm the identity of the RMF, as well as investigate intra-species genetic variation, 30 Knott’s-positive blood samples containing microfilariae that were morphologically consistent with *R. andersoni* were selected for molecular analysis. DNA was extracted using the ZR Fecal DNA Kit (Zymogen, Irvine, CA, USA) according to the manufacturer’s instructions. Nuclease-free water served as the DNA extraction control. RMF DNA was amplified using a previously described semi-nested PCR protocol targeting the first internal transcribed spacer region (ITS-1) of the rRNA gene cluster [22]. The primers FL1-F (5′-TTC CGT AGG TGA ACC TGC-3′) and FL2-R (5′-ATA TGC TTA AAT TCA GCG GG-3′) were used in the primary PCR reaction; the primers FL1-F and Di58S660 (5′-ACC CTC AAC CAG ACG TAC-3′) were used in the secondary PCR reaction [22]. DNA from a morphologically-identified adult *R. andersoni* nematode (RA-F3) isolated from a Finnish reindeer and nuclease-free water served as the positive and negative PCR controls, respectively. The PCR products were separated by agarose gel electrophoresis and viewed under UV light. PCR amplicons around 600 bp were excised using a clean razor blade and the PCR product purified using the QIAquick Gel Extraction Kit (Qiagen, Valencia, CA, USA). The ITS-1 PCR product was then cloned into a pGEM-T Easy vector (Promega, Madison, WI, USA) and transformed into competent DH5α *Escherichia coli* cells (Invitrogen, Grand Island, NY, USA) via a 40-second heat shock at 42 °C. Transformed cells were cultivated in S.O.C. medium (Life Technologies, Grand Island, NY, USA) for 1.5 h at 30 °C with shaking. The transformed cells (100 μl) were then plated on Luria broth agar plates containing 1 μg/ml carbenicillin and 100 μl ChromoMax IPTG/X-gal solution (Thermo Fisher Scientific, Waltham, MA, USA) as a top dressing. Cultures were incubated 24–48 h at 30 °C; single, white colonies were selected with a sterile toothpick and grown overnight in 5 ml Luria broth with 1 μg/ml carbenicillin. Cultures were centrifuged and the supernatant removed. Plasmids were purified from the remaining cell pellet using the Qiagen Spin Miniprep Plasmid Kit (Qiagen) following the manufacturer’s instructions. To confirm the presence of the filarioid ITS-1 PCR product insert, the plasmids were digested with EcoR1 restriction enzyme (Thermo Fisher Scientific) and examined via gel electrophoresis for multiple bands. Plasmids containing an insert of approximately 600 bp were sequenced at the University of Tennessee’s Genomics Core (Knoxville, TN, USA).

### Phylogenetic analysis of *R. andersoni* sequences

All 18S and ITS-1 consensus sequence chromatograms were trimmed and edited by hand using Sequencher 5.3 (Gene Codes Corporation, Ann Arbor, MI, USA). Edited sequences were compared against the few known sequences for filarioid nematodes from cervid hosts in the NCBI GenBank database. Genetic data was also compared with sequences obtained from adult reference nematodes (Table 1), which had been identified morphologically and subjected to DNA extraction and PCR amplification in our laboratory, as described above. Alignment and construction of neighbor-joining trees of ITS-1 and 18S filarioid worm sequences was performed using MEGA 6.0 [23]. All consensus sequences were deposited in the GenBank database under accession numbers KT020850, KT031392–KT031393; KT873719–KT873733; KT878970–KT878979l; and KU757075–KU757077 (see Tables 1 and 3 for details).

**Table 1:** Reference nematodes used in molecular analysis of blood samples obtained from cervids

Isolate	Species	Geographical origin	Host species	DNA target	GenBank accession no.
RA-F124	*Rumenfilaria andersoni*	Finland	*Rangifer tarandus*	18S	KT878978
RA-F113	*Rumenfilaria andersoni*	Finland	*Rangifer tarandus*	18S	KT878977
RA-F128	*Rumenfilaria andersoni*	Finland	*Rangifer tarandus*	18S	KT878979
RA-F3	*Rumenfilaria andersoni*	Finland	*Rangifer tarandus*	ITS-1	KT873731
ES-WY11	*Elaeophora schneideri*	Wyoming, USA	*Alces alces*	18S	KT031392
ES-WY50	*Elaeophora schneideri*	Wyoming, USA	*Alces alces*	ITS-1	KT873732
ES-CA1	*Elaeophora schneideri*	California, USA	*Rusa unicolor*	18S	KT020850
OC-AK1	*Onchocerca cervipedis*	Alaska, USA	*Alces alces*	18S	KT031393
SY-AK1	*Setaria yehi*	Alaska, USA	*Alces alces*	18S	KT878970
SY-GA3	*Setaria yehi*	Georgia, USA	*Odocoileus virginianus*	ITS-1; 18S	KU757075; KT878972

Adult nematodes were identified based on morphological characteristics. Geographial origin and host species refer to the place and host from which the nematode was isolated. DNA target refers to the targeted gene sequence (18S rRNA or ITS-1) that was amplified

## Results

### Prevalence of *R. andersoni* in free-ranging cervids of Minnesota and other U.S. states

During 2012–2015, RMF occurred in 20.5 % (95 % confidence interval [CI]: 16.3–24.7 %; *n* = 352) of Minnesota moose (Table 2). Presence of RMF among the Minnesota moose was independent of host gender (*χ*^2^ = 3.879, *df* = 1, *P* = 0.049) and age (Fisher’s exact test, *P* = 0.104). RMF were also detected in Minnesota white-tailed deer, with an overall prevalence of 5.6 % (95 % CI: 0–13.1 %; *n* = 36), but no RMF were detected in the Minnesota elk samples (0 %, *n* = 14) (Fig. 1a). Over the 4-year sampling period, RMF prevalence in the Minnesota moose varied slightly, ranging from 22.5 % (95 % CI: 16.0–29.0 %; *n* = 160) in 2013 to 14.3 % (95 % CI: 5.1–23.5 %; *n* = 56) in 2015, but differences among sampling years were not significant (Fisher’s exact test, *P* = 0.607) (Fig. 1b).

**Table 2:** Demographics of Minnesota moose sampled for nematode microfilariae prevalence from 2012 to 2015

Year	Season	Sex			Age				% RMF-positive
		M	F	Unknown	< 1 year	1– < 2 years	≥ 2 years	Unknown	
2012	Autumn	62	4	1	1	4	59	3	19.4 (13/67)
2013	Winter	28	103	2	1	9	121	2	22.6 (30/133)
	Spring	4	10	1	1	3	10	1	26.7 (4/15)
	Summer	1	6	0	1	1	5	0	14.3 (1/7)
	Autumn	1	4	0	1	0	4	0	20.0 (1/5)
2014	Winter	14	42	1	0	4	50	3	21.1 (12/57)
	Spring	0	3	0	1	1	1	0	66.7 (2/3)
	Summer	1	3	0	1	0	3	0	25.0 (1/4)
	Autumn	3	2	0	0	2	3	0	0 (0/5)
2015	Winter	14	37	0	0	2	48	1	13.7 (7/51)
	Spring	1	2	0	0	1	1	1	0 (0/3)
	Summer	1	1	0	0	0	2	0	50.0 (1/2)

Seasons are defined as winter (December-February), spring (March-May), summer (June–August), and autumn (September-November)

*Abbreviations*: *F* female, *M* male, *RMF* *Rumenfilaria andersoni* microfilariae

RMF were also observed in all other surveyed moose populations, including those in Maine at 21.4 % (95 % CI: 0–42.9 %; *n* = 14), New Hampshire at 25.0 % (95 % CI: 3.8–46.2 %; *n* = 16), Montana at 28.8 % (95 % CI: 18.4–39.2 %; *n* = 73), Washington at 31.3 % (95 % CI: 8.6–54 %; *n* = 16), and Alaska at 40.7 % (95 % CI: 22.2–59.2 %; *n* = 27) (Fig. 1c). No statistically significant differences were detected among all populations, including Minnesota (Fisher’s exact test, *P* = 0.013). Moreover, we failed to observe RMF in any of the Alaskan caribou blood samples or in mule or white-tailed deer from Washington.

### Genetic comparison of *R. andersoni* isolates

To confirm the identity of the RMF observed in the Knott’s tests and to investigate the intra-species genetic variation of *R. andersoni*, partial filarial ITS-1 sequences from RMF-positive blood samples of 15 North American moose and one white-tailed deer were sequenced (Table 3). Moose isolates varied in geographical origin, with five from Montana, eight from Minnesota, and two from Maine. Attempts to amplify additional isolates were not successful. Sequences obtained from a morphologically confirmed *R. andersoni* adult nematode (RA-F3) isolated from a Finnish reindeer served as the standard.

**Table 3:** *Rumenfilaria andersoni* ITS-1 target DNA sequences amplified from cervid blood*.* Unless otherwise indicated, isolates were obtained from moose (*Alces alces*)

Isolate	Geographical origin (USA)	GenBank accession no.
RA-MT4	Montana	KT873721
RA-MT8	Montana	KT873724
RA-MT31	Montana	KT873720
RA-MT43	Montana	KT873722
RA-MT44	Montana	KT873723
RA-MN1	Minnesota	KU757076
RA-MN2	Minnesota	KT873733
RA-MN3	Minnesota	KT873727
RA-MN4	Minnesota	KT873728
RA-MN5	Minnesota	KT873729
RA-MN6	Minnesota	KT873730
RA-MN7	Minnesota	KU757077
RA-MN9^a^	Minnesota	KT873719
RA-ME1	Maine	KT873726
RA-ME2	Maine	KT873725

^a^Host was a white-tailed deer (*Odocoileus virginianus*)

**Fig. 2:**
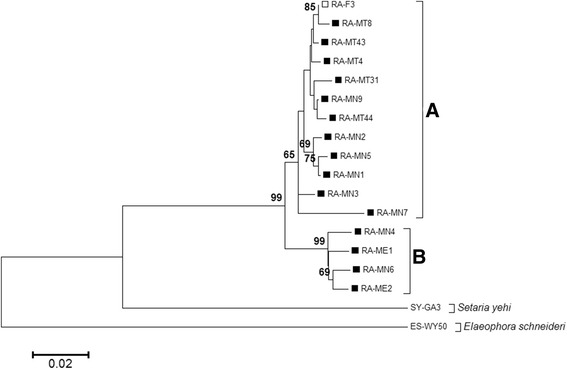
Phylogenetic analysis of ITS-1 sequences obtained from RMF-positive blood samples from cervid hosts. Sequences of 609 base pairs were aligned using ClustalW, and the evolutionary history was inferred using the Neighbor-Joining method. Evolutionary distances were computed using the Kimura 2-parameter method. The tree is drawn to scale. Bootstrap values (×1000) greater than 50 % are shown above the branches. RMF isolates are marked with solid boxes; ITS-1 Clades A and B are labelled. RA-F3 (*Rumenfilaria andersoni*; open box), *Setaria yehi*, and *Elaeophora schneideri* serve as reference standards. GenBank accession numbers for all isolates are listed in Tables 1 and 3

Overall, the RMF ITS-1 sequences were AT-rich, with multiple sections of repeats with variations in length between isolates. Phylogenetic analysis revealed that all of the ITS-1 sequences obtained from the RMF-positive blood clustered with the RA-F3 standard, branching into two distinct *R. andersoni* lineages (Fig. 2). The Minnesota isolates had representatives clustering into both clades, which we simply denoted as Clades A and B. All Montana isolates and six Minnesota isolates clustered into Clade A, which also contained the RA-F3 standard. Clade B contained both Maine isolates and two Minnesota isolates. Isolates of Clades A and B had a mean difference of 0.038 (SE = 0.007) base substitutions per base pair. Within clades, isolates had a mean difference of 0.015 (Clade A, SE = 0.003; Clade B, SE = 0.004) base substitutions per base pair for both Clades A and B.

### Identification of the filarioid *Setaria yehi* in Minnesota moose

**Fig. 3:**
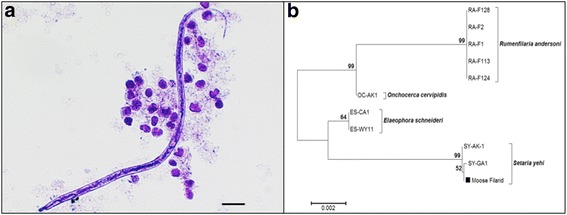
Morphological and genetic characterization identifies moose filarioid as *Setaria yehi*. **a** Image of unidentified microfilaria observed in blood from Minnesota moose. Sample was stained with methylene blue and image taken under a bright light microscope at 200× magnification. *Scale-bar*: 20 μm. **b** Phylogenetic comparison of 18S rRNA sequences (796 base pairs) from unknown filarioid (*black box*) and other known filarioid parasites of ungulates, with history inferred using the Neighbor-Joining method and evolutionary distances computed using the Kimura 2-parameter method. Tree is drawn to scale. Bootstrap values (×1000) are shown above branches. GenBank accession numbers for all isolates are listed in Tables 1 and 3

In addition to RMF, another morphologically distinct group of microfilariae was observed in 1.4 % (5/352) of Minnesota moose. These microfilariae were characterized by blunt, rounded heads and long, thinly tapered tails, measuring between 285 and 315 μm long and 5–7.5 μm wide (Fig. 3a). Dual infection with RMF and non-RMF microfilariae was observed in a single Minnesota moose. To identify this unknown filarioid, we attempted to sequence a portion of the 18S rRNA gene using nematode-wide primers from the five positive Minnesota moose blood samples [24]. Only one blood sample was successfully PCR amplified. A comparison of 796 base pairs from the unknown filarioid and 18S sequences from our reference nematodes (Table 1) revealed the unknown filarioid most closely resembled *Setaria yehi* (Spirurida: Onchocercidae) [25] (Fig. 3b).

## Discussion

Prior to the conclusion of this study, little was known about the occurrence of *R. andersoni* in cervid hosts. Knowledge about the distribution of *R. andersoni* was limited to the original description of this nematode in moose of Ontario [13] and was only recently expanded to include Alaska [14] and Finland [15]. Our survey of moose herds for *R. andersoni* suggests the geographical range of this filarioid nematode is much more extensive than was previously appreciated. RMF were detected in all of the wild moose herds we sampled, including herds geographically isolated from one another (Fig. 1c). These results imply that *R. andersoni* nematodes are widely distributed throughout the North American moose range and support previous studies suggesting moose might serve as the main reservoir host of *R. andersoni* [13, 16]. Interestingly, Alaskan moose had a higher RMF prevalence (40 %; *P* = 0.013) compared to other moose herds surveyed; however, this is not entirely unexpected as previous reports have shown *R. andersoni* prevalence in Alaska as high as 70 % [14]. Variations in vector and, or host density could potentially play an important role in *R. andersoni* prevalence and may be contributing to this disparity. It is possible that *R. andersoni* and/or its preferred vector may be more highly adapted to subarctic climates, proliferating more easily in a colder environment. The recent rapid expansion of *R. andersoni* into the subarctic regions of Finland [15, 16] supports this hypothesis, but additional evidence is needed to substantiate this claim. Further research on moose densities, vector distribution and competency, and distribution of other possible definitive hosts of *R. andersoni* will be useful in understanding if the parasite is more adapted to subarctic climates or if it is strictly a host and/or vector density-dependent mechanism.

Surveys for RMF in other cervid species revealed *R. andersoni* occurs in white-tailed deer, and deer can serve as a definitive host for the filarioid (Fig. 1a). Presence of RMF in white-tailed deer was documented once before in 2005 while investigating the emergence of *R. andersoni* in Finnish semi-domesticated reindeer [16]. The authors observed 22 % of the deer surveyed in Finland were RMF-positive, higher than the 5.6 % prevalence value observed in deer from Minnesota. As mentioned above, it is possible the parasite is better adapted to subarctic environments, thus explaining the lower parasite prevalence at the more southern latitude; however, a larger sample size along a latitudinal gradient would be needed to properly address that particular hypothesis.

In addition to white-tailed deer, our survey included specimens from caribou, elk, and mule deer from various geographical locales. We were unable to find evidence of *R. andersoni* infections within any of these species. This was surprising, as *R. andersoni* was found to be a common and abundant parasite in reindeer of Finland, with prevalence as high as 90 % in some locations [16]. Although the caribou and reindeer herds from Alaska and Finland are different subspecies, and it is possible that Alaskan caribou may be genetically more resistant to *R. andersoni* infection, we suspect the absence of *R. andersoni* in our Alaskan caribou specimens may be due to an insufficient sample size rather than a lack of host-parasite co-adaptation. Additionally, the number of elk and mule deer surveyed may have been insufficient, resulting in no RMF detected in the modified Knott’s test; however, it is also possible these species are not suitable hosts for the nematode. Further sampling will better characterize *R. andersoni* host range and suitability among North American cervid populations.

It should be noted that the use of the modified Knott’s test to estimate prevalence based on finding microfilariae in blood samples could result in an underestimation of the true prevalence of *R. andersoni* in their mammalian hosts. Timing of sample collection could significantly influence estimated prevalence values, potentially causing early-stage infections to be missed or causing false negatives with the synchronous influx of microfilariae into general circulation that coincides with circadian rhythms [16]. Furthermore, the modified Knott’s test is unable to detect infections with nematodes of a single sex due to the female filarioid being unable to mate and produce progeny. At this time, the only alternative method to estimate prevalence would be to grossly examine host carcasses for the presence of adult *R. andersoni*, presumably in the lymphatic vessels of the rumen; however, in addition to being a laborious and tedious process, fresh wildlife carcasses can be difficult to obtain.

To gain a better understanding of the diversity and genetic variation of *R. andersoni* nematodes, we compared ITS-1 sequences obtained from RMF-positive blood samples (Fig. 2). Our phylogenetic analysis suggests there are two clades of *R. andersoni* in North America, with all Montana isolates associating with Clade A and all Maine isolates falling into Clade B. Interestingly, Minnesota isolates have representatives in both clades. Possible reasons for the mixed clades in Minnesota include potential overlapping RMF populations or previous cervid translocation events. It is also possible multiple paratypes of *R. andersoni* circulate within the Minnesota cervid host populations. Previous morphological analyses of *R. andersoni* specimens revealed nematodes often differ by the number and arrangement of caudal papillae present [13, 15]. Combined, our ITS-1 data and the morphological descriptions suggest at least two separate *R. andersoni* populations exist; however, a dual DNA-based and adult morphological study will be needed to identify if there is a distinct relationship between caudal papillae phenotypes and phylogenetic assortment. Furthermore, studies comparing *R. andersoni* genotypes in the vector and cervid hosts would provide additional insight into *R. andersoni* population dynamics and may help to identify factors driving *R. andersoni* transmission and maintenance within the environment.

Here, we also observed our *R. andersoni* reference nematode’s (RA-F3) ITS-1 sequence clustered into Clade A with isolates from Minnesota and Montana (Fig. 2). This is significant as RA-F3 was isolated from a reindeer in Finland, which recently experienced a significant expansion of *R. andersoni* in wild and domestic reindeer herds [16]. Researchers hypothesized the colonization of Finnish cervids with *R. andersoni* resulted from the introduction of non-native white-tailed deer from North America, specifically the U.S. state of Minnesota. Five deer, one male and four females, were imported into southern Finland in 1935 as a gift from Finnish immigrants from northern Minnesota [26]. If Finnish *R. andersoni* nematodes’ ancestors originated from Minnesota, we would expect the Finnish ITS-1 sequences to be similar to those of North American specimens, especially those of Minnesota; conversely, we would expect significant genetic variation if the Finnish population had an extended history of geographical distribution and isolation in Fennoscandia. Our data demonstrate a lack of divergence between these isolates, indicating that a more recent, anthropogenically-driven introduction of the parasite occurred, supporting the Minnesota theory, rather than an introduction coinciding with the geographical colonization by moose from Central Europe and Russia after the last glaciation, approximately 10,000 years ago [27].

In addition to the presence of RMF, our data revealed another filarioid circulating within the Minnesota moose herd (Fig. 3). Sequencing and phylogenetics confirmed the identity of this filarial nematode to be *S. yehi*, a common parasite of cervids in North America (Fig. 3b). Adult *S. yehi* are most commonly found in the abdominal cavity and produce microfilariae that circulate within the bloodstream [14, 28]. Various mosquito genera are considered to be the major vectors of this nematode [29]. Although not much is known about the distribution of *S. yehi* in moose, previous reports documented *S. yehi*-infected moose in Alaska [30], Alberta [31], Ontario [32], and Wisconsin [30], suggesting *S. yehi* is likely widespread amongst North American moose herds. This parasite can cause a wide range of disease in cervids. Mild fibrin formation on serosal surfaces has been reported in *S. yehi* infected white-tailed deer and chronic peritonitis has been reported in infected Alaskan reindeer [33]. Additionally, intense inflammation, secondary bacterial peritonitis and mortality in nine free-ranging moose calves were attributed to migrating *S. yehi* and high intensities of microfilariae [14]. However, to our knowledge there have been no reports of clinical infections in adult moose, and the incidence of morbidity and mortality associated with *S. yehi* infections within moose populations have not been studied. Thus, it is unknown the impact *S. yehi* infections may have on moose population dynamics.

Although other nematode parasites are known to contribute to moose morbidity and mortality [6], our study was unable to establish a clear association between prevalence of *R. andersoni* and declining populations of moose. No significant difference in RMF prevalence was observed between the Minnesota moose herd, which is exhibiting a severe population decline [4], and those of Montana and New Hampshire, which are also decreasing [1, 3], albeit less dramatically; Washington, which has steadily increased in numbers since 1922 [34]; or Maine, where the moose population has increased over the last decade [35, 36] (Fig. 1c). Thus, prevalence of *R. andersoni* does not appear to have an obvious association with declining moose populations. At this time, it is still unknown what impact, if any, *R. andersoni* infection may have on the health of the moose host. Laaksonen et al., in 2010, observed macroscopic inflammatory changes within the ruminal lymphatic vessels of infected Finnish reindeer [15]; however, there have yet to be any reports of pathological changes associated with moose. Given the high prevalence of RMF in moose and the high prevalence and intensity of RMF in Finnish reindeer, future studies on potential subclinical and clinical disease is warranted. It is reasonable to hypothesize that infection with *R. andersoni* has a metabolic cost, and it is possible that heavy worm burdens or systemic microfilaremia would result in adverse health effects, potentially rendering the host more susceptible to other infectious agents or poor body condition, but future studies will be required to assess the validity of these hypotheses.

## Conclusions

*Rumenfilaria andersoni* is a nematode widespread throughout moose herds of North America. In addition to moose, white-tailed deer appears to be a natural, definitive host of this parasite. Recognizing the geographical distribution and host range of this filarioid is especially important for preventing the introduction of *R. andersoni* into naïve populations by translocation of animals by state conservation agencies or commercial hunting businesses. Our genetic comparison of *R. andersoni* isolates supports the hypothesis that the recent and rapid spread of *R. andersoni* in Finnish reindeer was due to the introduction of white-tailed deer from North America [15, 16], and further underscores the importance of a better awareness of *R. andersoni* biology. We were unable to correlate levels of parasite prevalence with moose population decline, and it is still unknown if *R. andersoni* infection can lead to clinical or subclinical disease. Continued efforts to document this parasite in cervid hosts will help to provide clarity on this topic.

## Abbreviations

CI, confidence interval; ITS-1, first internal transcribed spacer region of the rRNA gene; RMF, *Rumenfilaria andersoni* microfilariae
